# A New Model for Size-Dependent Tree Growth in Forests

**DOI:** 10.1371/journal.pone.0152219

**Published:** 2016-04-01

**Authors:** Masae Iwamoto Ishihara, Yasuo Konno, Kiyoshi Umeki, Yasuyuki Ohno, Kihachiro Kikuzawa

**Affiliations:** 1 Graduate School for International Development and Cooperation, Hiroshima University, Higashi-Hiroshima city, Hiroshima, Japan; 2 Department of Life Science of Agriculture, Obihiro University of Agriculture and Veterinary Medicine, Obihiro city, Hokkaido, Japan; 3 Graduate School of Horticulture, Chiba University, Matsudo city, Chiba, Japan; 4 Forestry Research Institute, Hokkaido Research Organization, Bibai city, Hokkaido, Japan; 5 Zushioku Onoecho, Yamashina, Kyoto city, Kyoto, Japan; Helmholtz Centre for Environmental Research - UFZ, GERMANY

## Abstract

Tree growth, especially diameter growth of tree stems, is an important issue for understanding the productivity and dynamics of forest stands. Metabolic scaling theory predicted that the 2/3 power of stem diameter at a certain time is a linear function of the 2/3 power of the initial diameter and that the diameter growth rate scales to the 1/3 power of the initial diameter. We tested these predictions of the metabolic scaling theory for 11 Japanese secondary forests at various growth stages. The predictions were not supported by the data, especially in younger stands. Alternatively, we proposed a new theoretical model for stem diameter growth on the basis of six assumptions. All these assumptions were supported by the data. The model produced a nearly linear to curvilinear relationship between the 2/3 power of stem diameters at two different times. It also fitted well to the curvilinear relationship between diameter growth rate and the initial diameter. Our model fitted better than the metabolic scaling theory, suggesting the importance of asymmetric competition among trees, which has not been incorporated in the metabolic scaling theory.

## Introduction

It is important to understand and predict tree growth because it is related to the productivity and dynamics of forest stands. Furthermore, tree growth rate is an important demographic parameter, together with mortality and recruitment rates [1]. Among various measurements of tree growth, diameter of tree stem has been most widely monitored because of the easiness and accuracy of the measurement compared to tree height.

There are several theoretical models to express stem diameter growth of individual tree. One of the simplest is a model based on the metabolic scaling theory. Enquist *et al*. [2] predicted that stem diameter growth rate scales to the 1/3 power of the diameter. This prediction was deduced from the assumption that growth rate of tree biomass is proportional to its gross photosynthetic rate, which in turn is determined by leaf biomass. This assumption means that both small and large trees have the same resource availability per unit leaf biomass.

However, resource availability may depend on tree size. In a closed forest stand, light is a limited resource and tree growth is governed mainly by light conditions [3–5]. Availability and competition for light is size-dependent and asymmetric [6–8]. Larger trees have an advantage over smaller trees, since the former shade the latter but the latter rarely do so [9]. In some extreme cases, competition is one-sided: larger tree suppress the growth of smaller trees but not vice versa [7,10,11]. Recent studies [6,12] extended the metabolic scaling theory to incorporate asymmetric competition. Their model fitted better to the data of natural forests than that of Enquist *et al*. [2], suggesting the importance of asymmetric competition.

Here, we propose a new theoretical model for tree diameter growth that implicitly considers asymmetric competition by extending the model of Kikuzawa [10]. Our model is based on a tree size distribution function. Frequency distribution of the tree biomass is often inverse J-shaped with many small trees and few larger trees. Small size differences among trees at the seedling or sapling stage due to genetic factors or slight differences in environmental conditions at the micro site will be amplified by competition among individuals. The initial normal frequency distribution in biomass becomes skewed to the right under asymmetric competition [7,11,13,14]. Such skewed size distribution can be expressed in *Y*-*N* curve: *Y* = *N*/(*AN* + *B*) [15] where *Y* is the cumulative tree biomass in the stand summed for the largest *N* trees and *N* is the rank of a tree ordered from the largest to the smallest tree. This relationship fitted to both even-aged plantation and uneven-aged natural forests [15,16]. Our model is based on this *Y*-*N* curve, which was later derived theoretically from a growth model assuming asymmetric competition [17]. We tested assumptions of our model and the fit of our model against tree growth data of multi-species secondary deciduous broadleaf forests in Hokkaido, northern Japan. We compared our model to the model by Enquist *et al*. [2].

## Materials and Methods

### Model of Enquist *et al*. (1999)

Enquist *et al*. [2] proposed a model for stem diameter growth on the basis of the metabolic scaling theory. They started from the assumption that whole plant biomass growth rate (d*M*/d*t*) is proportional to leaf biomass (*L*_B_)

$$\frac{dM}{dt}=C_{\text{G}}L_{\text{B}}$$ (1)

where *M* is whole plant biomass, *t* is time, *C*_G_ is a constant [18,19]. Here, leaf biomass *L*_B_ is proportional to the 3/4 power of individual tree volume *V* (= *M*/*ρ*; *ρ* being the density of tree body). On the other hand, stem diameter of a tree is proportional to the 3/8 power of the tree’s volume. By using these allometric correlations, Enquist *et al*. [2] obtained the following equation for stem diameter growth:

$$\frac{dD}{dt}=\left(\frac{3C}{2\rho }\right)D^{\frac{1}{3}}$$ (2)

where *C* is the proportionality constant. By substitution and integration from *t* = 0 to *t =* T, they derived the following equation:

$$D_{\text{T}}{}^{\frac{2}{3}}-D_{0}{}^{\frac{2}{3}}=\int_{0}^{\text{T}}\frac{C\left(t\right)}{\rho \left(t\right)}dt$$ (3)

The 2/3 power of stem diameter at time T (*D*_T_) can be expressed as a linear function of the 2/3 power of the initial stem diameter (*D*_0_), having a positive intercept and a slope of unity. Enquist *et al*. [2] argued that this equation was held in a secondary tropical forest.

### Alternative new model

We developed an alternative tree growth model by extending that of Kikuzawa [10]. This model is based on six assumptions that will be described in the following parts:

Assumption 1: *Y*-*N* curve fits to the size frequency distribution of trees.
Assumption 2: A tree’s rank does not change with time.
Assumption 3: Stand biomass follows a power function of the height of the highest individual in the stand.
Assumption 4: Allometric relationship between tree biomass and tree height.
Assumption 5: Parameter *B* scales with parameter *A* of *Y*-*N* curve.
Assumption 6: Individual tree biomass scales with the 8/3 power of stem diameter.

Hozumi *et al*. [15] observed in natural forests and a plantation that the size frequency distribution of trees is inverse J-shaped, with many small trees and few larger trees. In many cases [15,16], such size hierarchy can be expressed by the following equation (assumption 1):

$$\;Y=\frac{N}{AN+B}$$ (4)

where *Y* is the cumulative biomass in the stand summed from the individual with largest biomass to the *N-*th largest tree, *N* is the rank of a tree ordered from the largest individual, and *A* and *B* are parameters that are constants but change with time. Later, this equation was derived theoretically from the tree growth model that assumed asymmetrical competition [17]. Therefore, this relationship stands on a firm ground.

Under Eq (4), the biomass of an individual tree (*M*) that comprises the stand is expressed by the following equation [15]:

$$M=\frac{B}{{\left(AN+B\right)}^{2}}$$ (5)

This equation means that individual tree’s biomass is expressed as the function of *N*, the tree’s rank. Individual tree biomass of the same rank but different time (*t* = 0, T) will be expressed by the following equations:

$$M_{0}=\frac{B_{0}}{{\left(A_{0}N+B_{0}\right)}^{2}}$$ (5’)

$$M_{\text{T}}=\frac{B_{\text{T}}}{{\left(A_{\text{T}}N+B_{\text{T}}\right)}^{2}}$$ (5”)

Kikuzawa [10] suggested that under one-sided competition, individual tree’s rank changes little. Assuming that an individual tree’s rank does not change between two different times (assumption 2), we can eliminate *N* from Eqs (5’) and (5”) and obtain the following relationship between *M*_0_ and *M*_T_:

MT = BT(ATA0(B012M0−12−B0)+BT)2 (6)

Parameters *A* and *B* have biological meanings, and there is a relationship between parameters *A* and *B* [10]. Parameter *A* is a reciprocal of stand biomass (*Y*_max_) [15]:

$$A\;=\;1/Y_{max}$$ (7)

*Y*_max_ was assumed to be expressed as follows

$$Y_{max}\;=\;d\;H_{max}$$ (8)

where *d* is stand biomass divided by the height of the highest individual in the stand (*H*_max_). Kira and Shidei [20] define *d* as dry matter density and stated that *d* takes a constant value of 1–1.5 kg/m^3^, irrespective of stand height. By generalizing their findings, Kikuzawa [10] found that *d* is also a function of *H*_max_:

$$d=K_{1}H_{\text{max}}{}^{a}$$ (9)

where *a* is a parameter and *K*_1_ is a constant. When *a* = 0, Eq (9) will be consistent with the statement of Kira and Shidei [20]. Substituting Eq (8) by Eq (9) will yield the following power function between stand biomass and the height of the highest individual in the stand (assumption 3):

$$Y_{\text{max}}=K_{1}H_{\text{max}}{}^{a+1}$$ (10)

Substituting Eq (7) by Eq (10), we obtain

$$A=K_{1}{}^{-1}H_{\text{max}}{}^{-(a+1)}$$ (11)

Parameter *B* is the reciprocal of the maximum individual biomass of the stand (*M*_max_) [15]:

$$B\;=\;1/M_{max}$$ (12)

Kikuzawa [10] assumed that tree biomass *M* has an allometric relationship with tree height (*H*) (assumption 4) [21]:

$$M\;=\;K_{2}H^{b}$$ (13)

where *b* is a parameter and *K*_2_ is a constant. Applying this allometric relation to the maximum individual biomass (*M*_max_), we obtain:

$$M_{max}\;=\;K_{2}\;H_{max}{}^{b}$$ (14)

By applying Eqs (14) to (12), we obtain:

$$B=K_{2}{}^{-1}H_{\text{max}}{}^{-b}$$ (15)

From Eqs (11) and (15), the scaling relationship between *A* and *B* is obtained (assumption 5) for full-density stands as follows:

B=K3Ab(a+1)=K3Ac (16)

where *K*_3_ and *c* are constants.

Substitution of Eq (16)) in Eq (6) will yield the following:

MT=K3ATc[ATA0[(K3A0cM0)12−K3A0c]+K3ATc]2 (17)

This equation shows the relationship between the biomass of the same ranked tree at a different time under the condition that Eq (4) is equally held at different times.

West *et al*. [22] and Enquist *et al*. [2] showed that the biomass of an individual tree is proportional to the 8/3 power of stem diameter *D*, *M = K*_4_*D*^8/3^ (assumption 6). Eq (17) can be rewritten considering this allometric relationship as follows:

DT2/3=[(K3ATcK4)[ATA0[(K3A0c/K4)12(D02/3)−2−K3A0c]+K3ATc]2]14 (18)

This equation expresses the relationship between the 2/3 power of stem diameter at *t* = T to the 2/3 power of the initial stem diameter (*t* = 0) and may be an alternative to the prediction of Enquist *et al*. (Eq 3).

Moreover, the diameter growth rate, d*D*/*dt* ≈ (*D*_T_—*D*_0_)/*Δt*, can be obtained using Eq (18):

dDdt≈DT − D0Δt=[[(K3ATcK4)[ATA0[(K3A0cK4)12D0−43−K3A0c]+K3ATc]2]38−D0]/Δt (19)

By using this equation, we can examine the relationship between diameter growth rate and initial size (*D*_0_).

### Data set

To test the six assumptions of our model and the fit of our model (Eqs 18 and 19) and the model of Enquist *et al*. [2], we used data sets of 11 secondary deciduous broadleaf forests in Hokkaido, northern Japan (Table 1). Mean annual temperature and annual precipitation during past 30 years at the nearest meteorological stations of 11 forests were 5.5–7.3°C and 887–1415 mm, respectively. Permanent plots between 25 m^2^ and 2500 m^2^ in area were set. The number of trees per hectare ranged from 2,000 to 200,000 (Table 1). Stand age and maximum stem diameter in each plot were highly correlated (*r* = 0.978, *P*<0.001). Therefore, we used stand age and the maximum stem diameter as a surrogate of stand maturity and named the plots in order of maturity: plot 1 the youngest and plot 11 the most mature.

**Table 1:** Description of plots used for analyses.

Plot	Location	Lat.	Long.	Elev. (m)	Plot area (m^2^)	Age (yr)	DBH_max_ (cm)	H_max_ (cm)	S	1-D	N	Study years	Interval (yr)
P1	Ashibetsu	43° 37'	142° 05'	550	25	7–10	3.0	4.3	3	0.40	52	7	1
P2	Ashibetsu	43° 37'	142° 05'	550	25	7–10	3.7	5.4	4	0.10	75	8	1
P3	Ashibetsu	43° 37'	142° 05'	550	25	7–10	4.9	4.7	4	0.55	55	8	1
P4	Tohbetsu	43° 31'	141° 38'	370	100	10	7.0	10.0^†^	3	0.21	68	12	1
P5*	Tohbetsu	43° 31'	141° 38'	370	100	13	9.0	10.0	2	0.35	18	9	1
P6^¶^	Okoppe	44° 21'	143° 03'	200	100	20	9.5	12^‡^	16	0.85	139	15	2
P7	Okoppe	44° 21'	143° 03'	200	400	20	13.2	10^‡^	14	0.80	265	15	2
P8	Bibai	43° 16'	141° 52'	210	1000	20	15.5	15.0	14	0.68	389	7	2
P9	Okoppe	44° 17'	142° 57'	190	2000	62	26.6	20^‡^	13	0.83	141	15	2
P10^¶^	Okoppe	44° 17'	142° 57'	190	2000	62	35.7	20^‡^	18	0.79	232	15	2
P11	Mitsuishi	42° 20'	142° 43'	110	2500	<90	53.1	25^‡^	25	0.91	441	11	2

Lat.: latitude; Long.: longitude; Elev.: elevation; Age: estimated stand age at the initial census; DBH_max_: maximum stem diameter at breast height at the initial census; H_max_: maximum tree height at the initial census; S: number of species in the plot; D: Simpson’s diversity index; N: number of trees in the plot.

* Thinned plot.

^¶^ Largest tree that was assumed to have regenerated at a different time was excluded from the analyses.

^†^ Maximum tree height was measured in the third census.

^‡^Maximum tree height based on observation.

Plots 1 to 5 were young forests that naturally regenerated after scarifications, dominated by birch trees (*Betula ermanii* and *B*. *maximowiczii*) [10,23,24] (S1 Table). Plot 5 was artificially thinned. Plots 6, 7, 8, 9, and 10 were relatively mature mixed forests of birches and some other tree species such as oak (*Quercus crispula*), maple (*Acer mono* and *A*. *japonicum*), and others that naturally regenerated after forest fires. Plot 11 was the most mature secondary forest that had been regenerated after fuel wood production and composed of oak, *Carpinus laxiflora*, *Prunus sargentii*, and other tree species. A few conifer trees (*Abies sachalinensis*) grew in plot 10 and 11.

Stem diameters at breast height (1.3 m aboveground) were measured using a measuring tape or a caliper for a 1- or 2-year interval for 7 to 15 years. The position of diameter measurement was marked with paint. Tree height was measured only in plots 1–5 for all censused years. All trees in each plot were analyzed together, regardless of species, but trees that died during the census were excluded from the analyses. When *D*_T_ < *D*_0_, diameter growth (d*D*) was set as 0.

### Analysis

To test the equation of Enquist *et al*. [2] (Eq 3), we conducted reduced major axis (RMA) regression of the 2/3 power of the initial stem diameter (*D*_0_) to the 2/3 power of the diameter at final census *t* = T (*D*_T_). RMA regression, one of model II regression methods, was used because the relationship between *D*_0_ and *D*_T_ does not imply a direct causal relationship [25]. The lmodel2 function of R 2.15.3 was used to calculate the slope and intercept for RMA regression.

We tested the six assumptions used to derive our model. To test assumption 1 and 3–6, individual tree aboveground biomass was estimated by using an allometric equation. Although site- and species-specific allometric equations may give most accurate biomass estimates, we did not have an allometric equation for the studied forests and for each species. Recent studies [26,27] showed that generic multi-species equation created from compiled data is the best available choice when site-specific equation is not available. Thus, we decided to use a generic allometric equation created for natural forests in Japan [27]:

$$M\;=\;exp(-1.196\;+\;1.622\;\times \;ln(D)\;+\;0.338\;\times \;{\left(ln(D)\right)}^{2}\;-0.044\;\times \;{\left(ln(D)\right)}^{3}\;+\;0.708\;\times \;ln(\delta ))\;\times CF$$

where *M* is aboveground biomass of a tree, *D* is stem diameter at breast height, *δ* is species-specific wood density, and CF is the correction factor, which is 1.029 in this case. This equation was created from the compiled data of 1203 trees belonging to 102 species (60 deciduous angiosperm, 32 evergreen angiosperm, and 10 evergreen gymnosperm species) harvested from 70 natural forests all over Japan. This equation provided a similar biomass estimate as an allometric equation that had tree height as an additional predictive variable [27].

To test the assumption 1 (*Y*-*N* curve fits to the size-frequency distribution of trees), Eq (4) was fitted to the data by non-linear regression for each plot and each year via nls function of the R software.

To test the assumption 2 (a tree’s rank does not change with time), Spearman’s *ρ* was calculated between the rank at the initial census and that at the last census.

Assumptions 3 and 4 were tested in plots 1–5 where tree height was measured. In plots 6–11, tree height was too high to be measured accurately and only assumption 5 that was derived from the assumption 3 and 4 was tested. To test the assumption 3 (stand biomass follows a power function of the height of the highest tree in the stand), we calculated stand biomass by summing aboveground biomass for all trees in each plot for each census year. Log-transformed stand biomass was regressed to the log-transformed height of the highest individuals by ordinary least square (OLS) regression. To test the assumption 4 (allometric relationship between tree biomass and tree height), log-transformed aboveground biomass was regressed to log-transformed tree height by OLS regression for each plot and census year. Note that the above generic allometric equation used to estimate aboveground biomass [27] did not include tree height as a predictive variable, so the relationship between tree biomass and tree height, if it does exist, is not an artifact.

We tested the assumption 5 about the relationship between *A* and *B* by fitting ln (*B*) = ln (*K*_3_) + *c* × ln (*A*) to the parameter estimates of *A* and *B* obtained after fitting *Y*-*N* curve (assumption 1) by ordinary least square (OLS) regression and parameters *K*_3_ and *c* were obtained.

To test the assumption 6 (individual tree biomass scales with the 8/3 power of stem diameter), ln (*M*) = ln (*K*_4_) + *e* × ln (*D*) was fitted to log-transformed tree aboveground biomass (*M*) and log-transformed stem diameter (*D*) by RMA and OLS regressions. Because *M* was estimated from the allometric equation, which is the polynomial function of stem diameter, the scaling of *M* to *D* may be an artifact. Therefore, we also tested this assumption by using another dataset that had been used to create the generic allometric equation in Japan [27,28]. The dataset included measured aboveground biomass and stem diameter of 1203 trees destructively harvested from 70 boreal, temperate, and subtropical forests in Japan (hereafter called harvested dataset). This dataset was also used to additionally test assumption 4.

Using the estimates of parameters *A*_0_, *A*_T_, *K*_3_, *K*_4_ and *c*, we tested the fit of Eqs (18) and (19) to the 11 plots. We did not fit these equations directly to the data because these equations are highly flexible and can generate various curves depending on the five parameters. Thus, we first obtained the plot-specific estimates of *A*_0_, *A*_T_, *K*_3_, and *c* through tests of assumptions 1 and 5 (S2 Table). For *K*_4_, we did not use plot-specific estimate because it seemed to be affected by artifact (see previous paragraph). Instead, we used a common value that was estimated from harvested dataset by fitting ln (*M*) = ln (*K*_4_) + 8/3 × ln (*D*). Then, we fitted Eqs (18) and (19) using these fixed parameter values.

## Results

### Evaluation of the model of Enquist *et al*. (1999)

**Fig 1:**
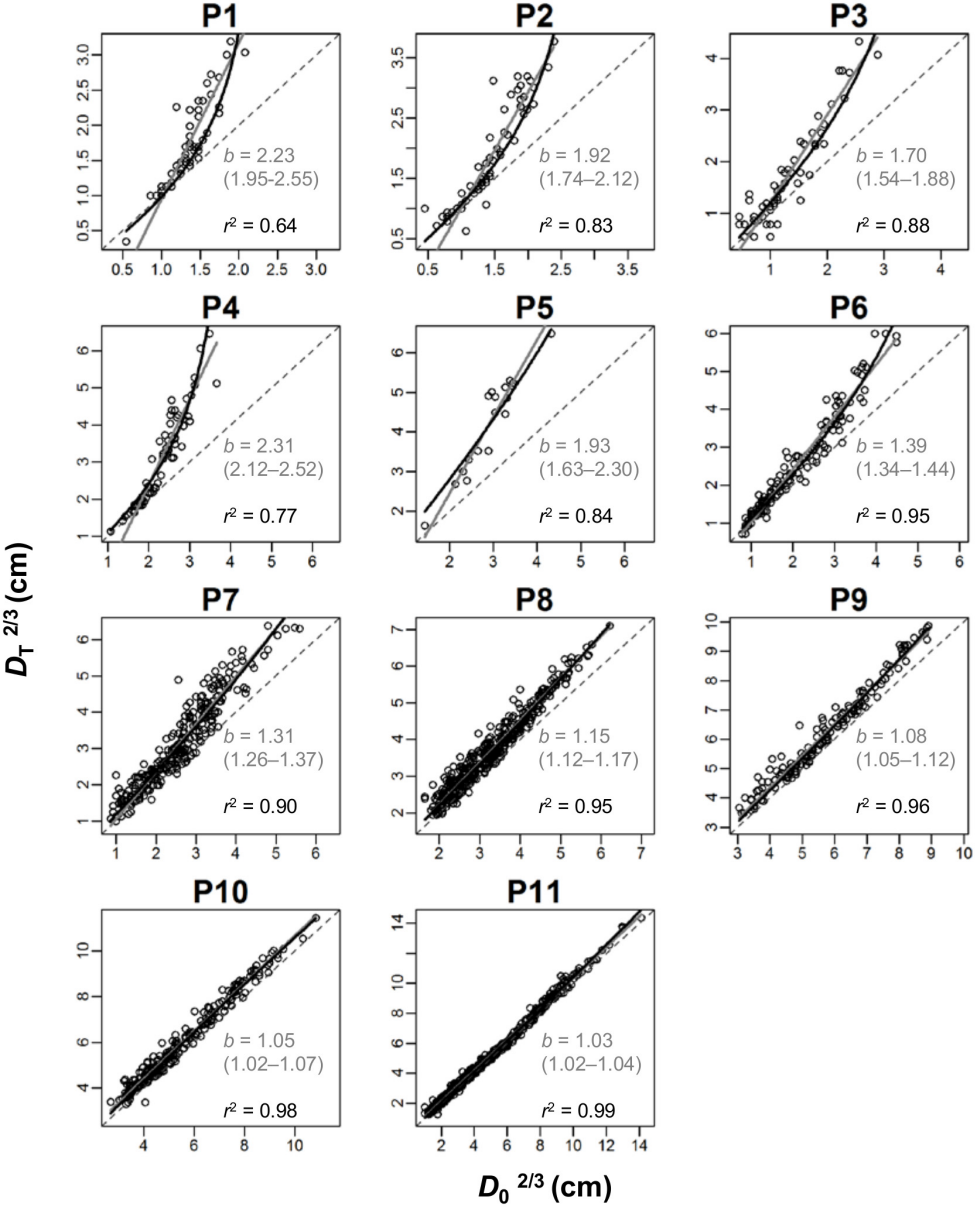
Relationship between the 2/3 power of initial stem diameter (*D*02/3) and final diameter (*D*T2/3) in 11 forests. Gray lines are *D*_T_^2/3^ = *a* + *bD*_0_^2/3^ fitted by the reduced major axis regression. Estimated values of *b* are shown with the 95% confidence interval. Black curves show the relationship predicted by alternative model expressed in Eq (18) with adjusted *r*^2^ values. For the values of parameters of alternative model, see S2 Table. Dashed lines show 1:1. Plot 1 is the youngest and plot 11 is the most mature forest.

Eq (3) proposed by Enquist *et al*. [2] fitted well only to some mature stands. Fig 1 shows the relationship between the 2/3 power of the initial diameter (*D*_0_^2/3^) and that of the final diameter (*D*_T_^2/3^). For example, in plot 11, the estimated slope of RMA regression was 1.03 (95% confidence interval [CI] = 1.02–1.04), which was almost identical to the unity expected by Eq (3). However, in younger stands, the relationship between *D*_0_^2/3^ and *D*_T_^2/3^ was not approximated well by a line. If we approximated by a line, for example in plot 4, the slope was obviously greater than unity and the intercept was negative (*D*_T_^2/3^ = -2.22 + 2.31*D*_0_^2/3^; *r*^2^ = 0.89).

The value of slope *b* in the equation *D*_T_^2/3^ = *a* + *bD*_0_^2/3^ had a clear relationship with stand maturity. Slope *b* was near unity in the mature stands with large trees (plots 9–11, Fig 1), while it was greater than 1 and was near 2 in the younger stands with small trees (plots 1–5). Intermediate-aged stands (plots 6–8) showed intermediate *b* values between 1 and 2. Thus, the model of Enquist *et al*. [2] was not supported in small-sized young stands. The result did not change even when OLS regression was used instead of RMA regression (data not shown).

### Assumption 1: *Y*-*N* curve fits to the size frequency distribution of trees

**Fig 2:**
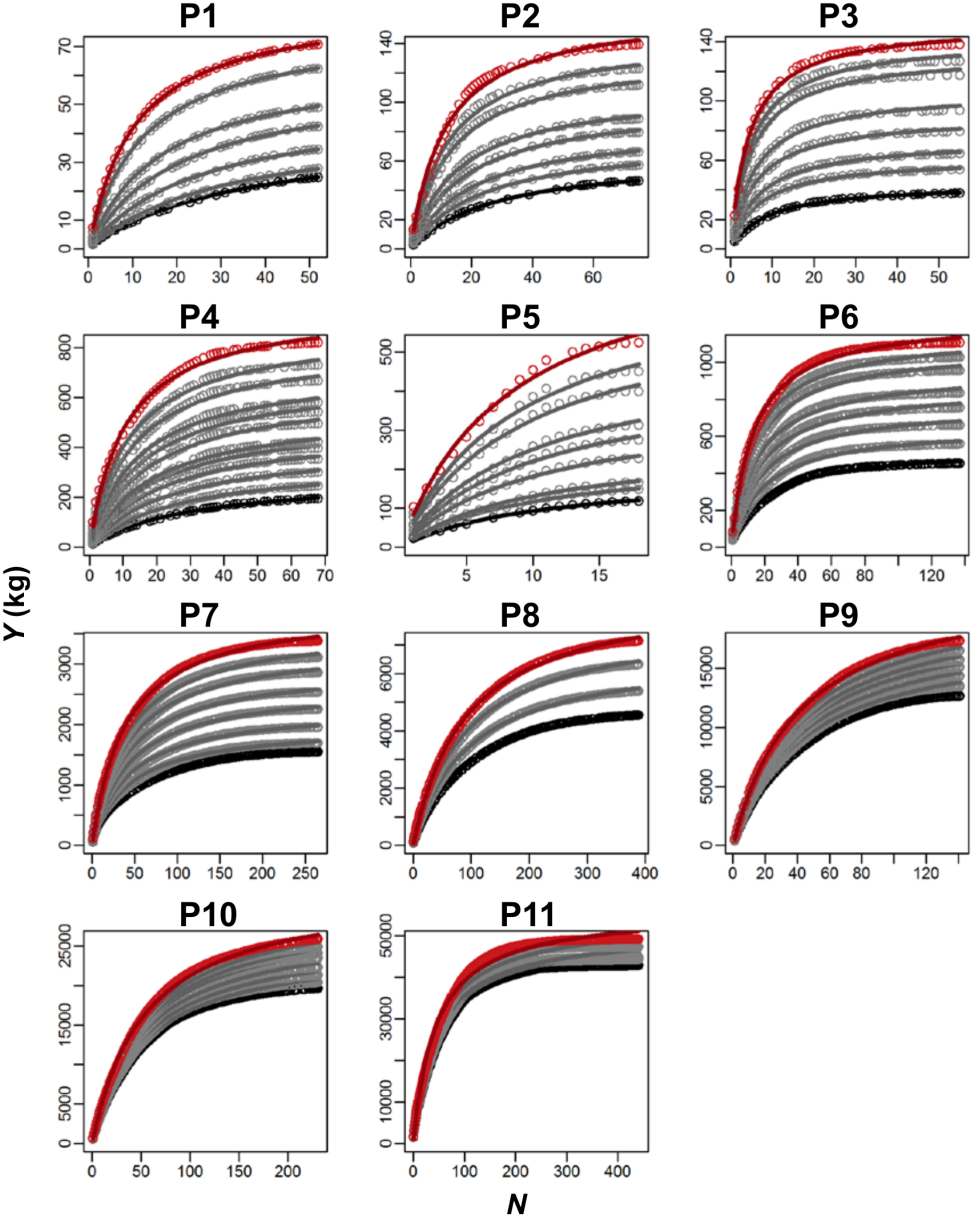
Fit of *Y*-*N* equation (Hozumi *et al*. 1968) in 11 forests. *Y* is the cumulative aboveground biomass summed for *N* largest trees and *N* is the cumulative number of trees from the largest tree (i.e. tree’s rank). Curves are fitted *Y*-*N* equations, *Y* = *N* / (*AN* + *B*). Data and fitted *Y*-*N* curve of the first year census (black), those of last census (red), and the rests (gray). Plot 1 is the youngest and plot 11 is the most mature forest.

*Y*-*N* curve between cumulative biomass in the stand summed from the largest tree to the *N*-th largest tree (*Y*) and tree’s rank (*N*) fitted well to the actual size structure in all stands (Fig 2).

### Assumption 2: Tree’s rank does not change with time

Individual tree’s rank at the initial census and that at the final census were strongly correlated (Spearman’s *ρ* = 0.903–0.996, *P*<0.001). Therefore, we can assume no or few changes in a tree’s rank with time.

### Assumption 3: Stand biomass follows a power function of the height of the highest individual in the stand

**Fig 3:**
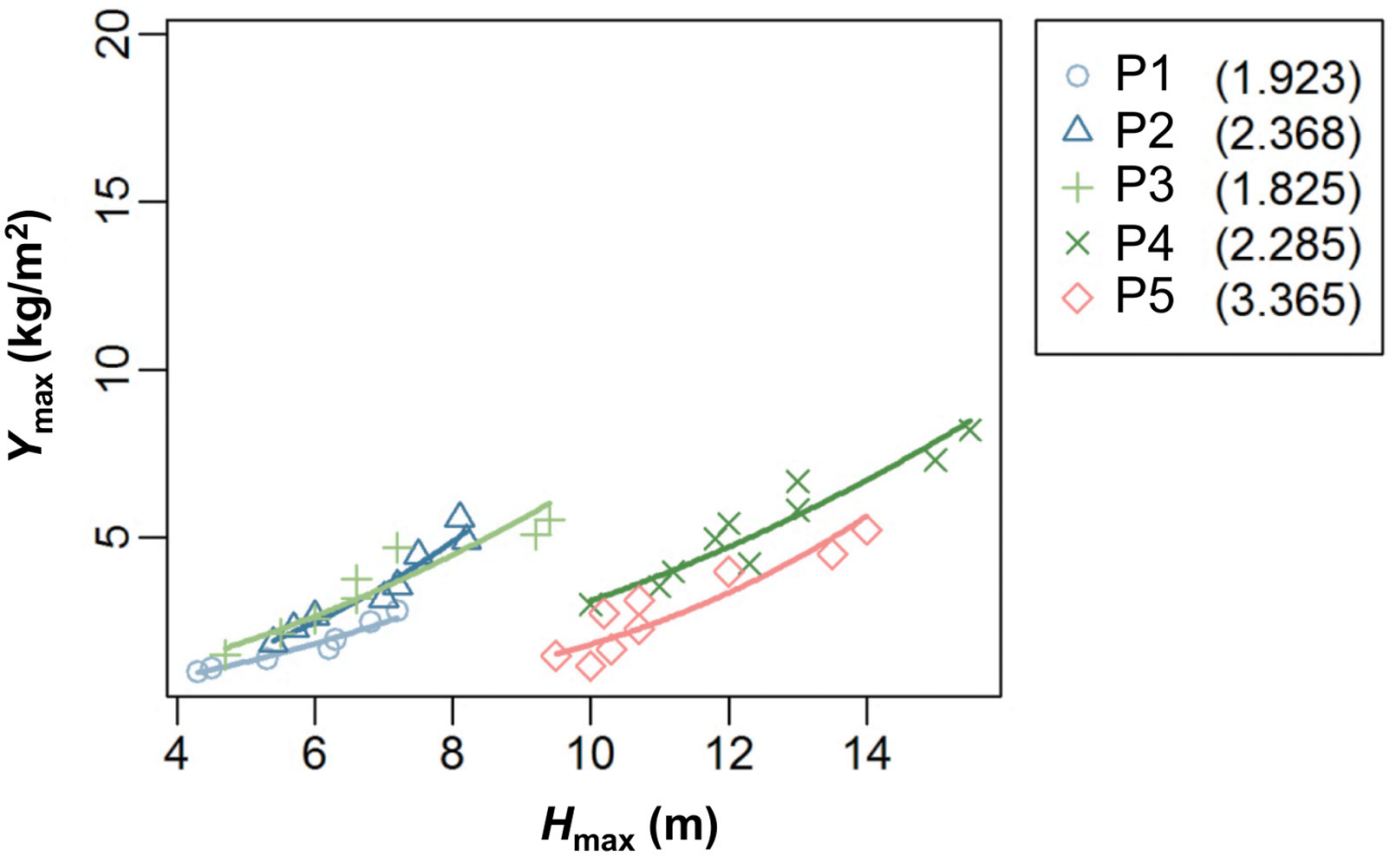
Relationship between stand biomass (*Y*_max_) and the height of the highest individual in the stand (*H*_max_). Regression curves are *Y*_max_ = *K*_1_ *H*_max_^a+1^ fitted for 7–12 year-data in each plot. The estimated values of *a*+1 are shown in the legend (figures in parentheses). Plot 5 showed smaller biomass due to stand thinning.

In plots 1–5 where tree height was measured, stand total biomass was well expressed by *Y*_max_ = *K*_1_ *H*_max_^a+1^ (Eq 8, Fig 3, *P* < 0.002, adjusted *r*^2^ = 0.74–0.95). The 95% CI for *a* + 1 was larger than 1 in all five plots. Therefore, assumption 3 was supported for all the five plots.

### Assumption 4: Allometric relationship between tree biomass and tree height

**Fig 4:**
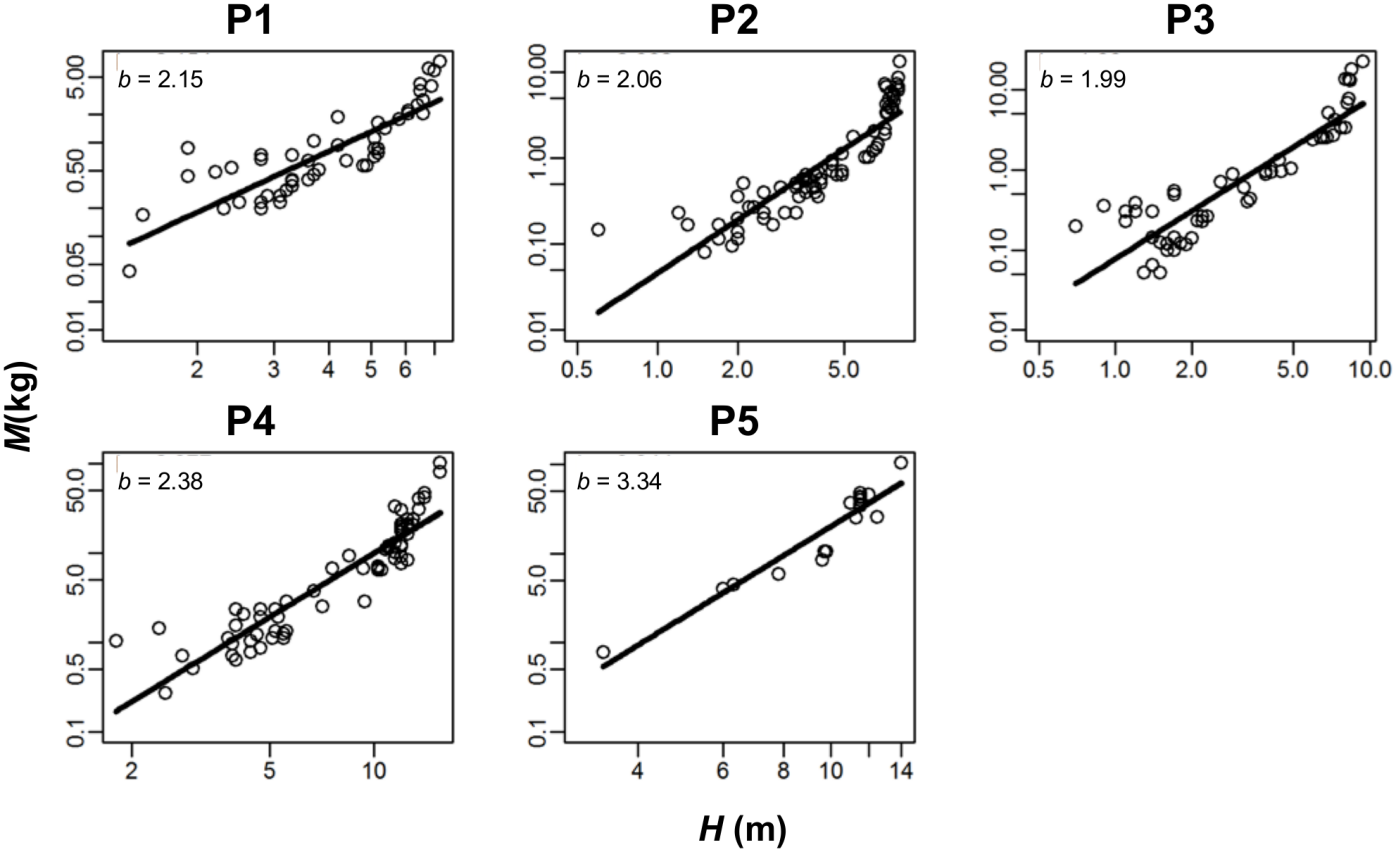
Allometric relationship between tree aboveground biomass (*M*) and tree height (*H*) in the last-year census. Regression lines are ln(*M*) = ln(*K*_2_) + *b* × ln(*H*) fitted for last-year data. The estimated values of *b* are shown.

Although the generic allometric equation used to estimate aboveground biomass did not have tree height as a predictive variable, estimated aboveground biomass showed an allometric relationship to tree height in plots 1–5 in each year, i.e., *M* = *K*_2_ *H*^*b*^, with *b* = 1.29–3.68 significantly different from 0 (Fig 4, *P* < 0.0001, adjusted *r*^2^ = 0.56–0.95, except *r*^2^ = 0.24 for 1 year in plot 1). Measured tree biomass of harvested dataset [27] also showed an allometric relationship to tree height (*P* < 0.0001, adjusted *r*^2^ = 0.89, *b* = 3.28). Therefore, assumption 4 was supported for all five plots and for the compiled harvested dataset.

### Assumption 5: Parameter *B* scales with *A*

**Fig 5:**
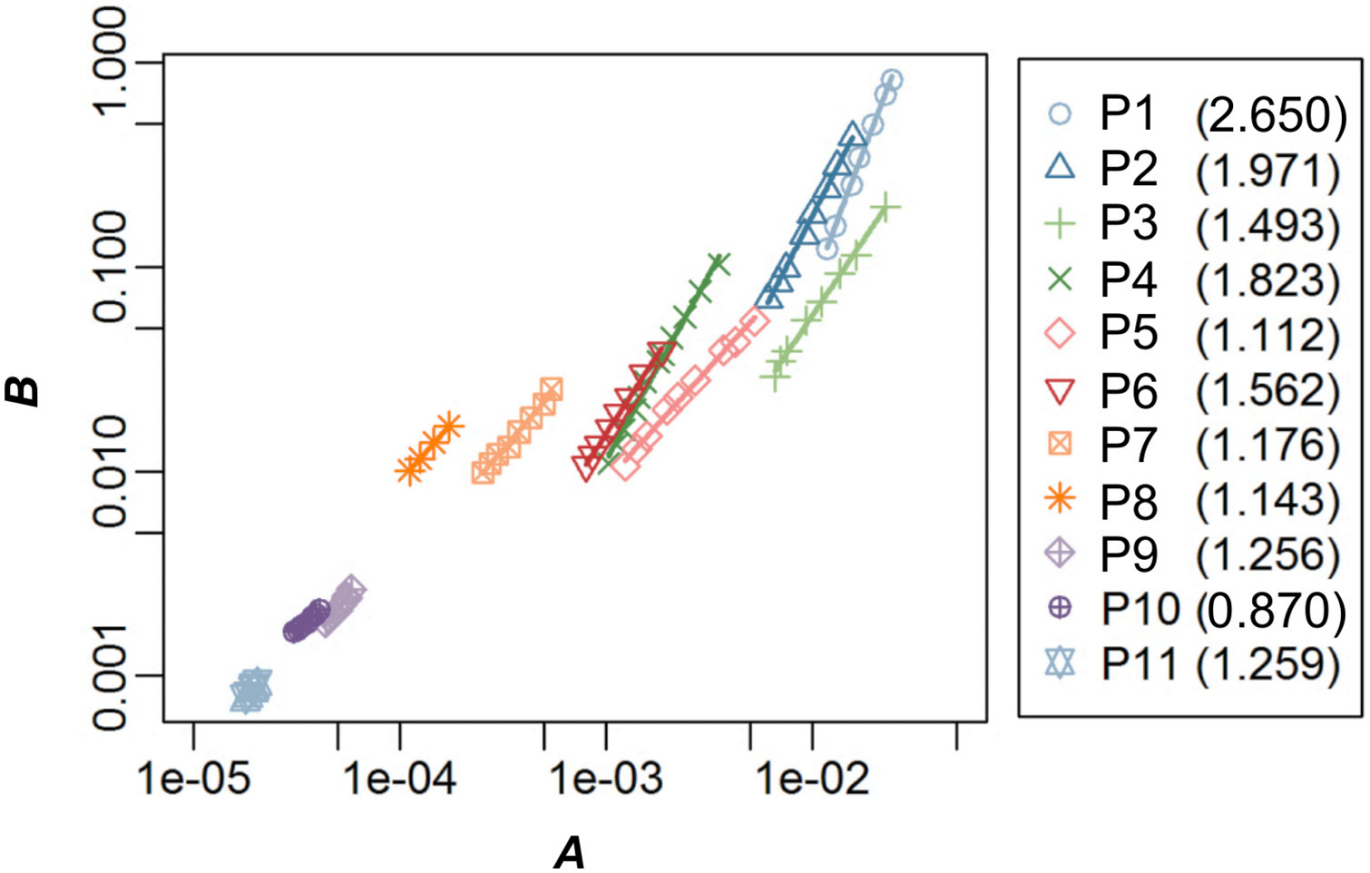
Logarithmic relationship between parameters *A* and *B* of *Y*-*N* equation. Regression lines are ln(*B*) = ln(*K*_3_) + *c* ×ln(*A*) fitted for 7–15-year data censused in each of 11 plots. The estimated values of *c* are shown in the legend (figures in parentheses). Note that axes are in logarithmic scales. Plot 1 is the youngest and plot 11 is the most mature forest.

Estimated parameters *A* and *B* of the *Y*-*N* curve followed the power function *B* = *K*_3_*A*^c^ in all plots, with parameter *c* significantly different from 0 (Fig 5, *P* < 0.0001, adjusted *r*^2^ = 0.98–1.00). Therefore, assumption 5 was supported.

### Assumption 6: Individual tree biomass scales with the 8/3 power of stem diameter

**Fig 6:**
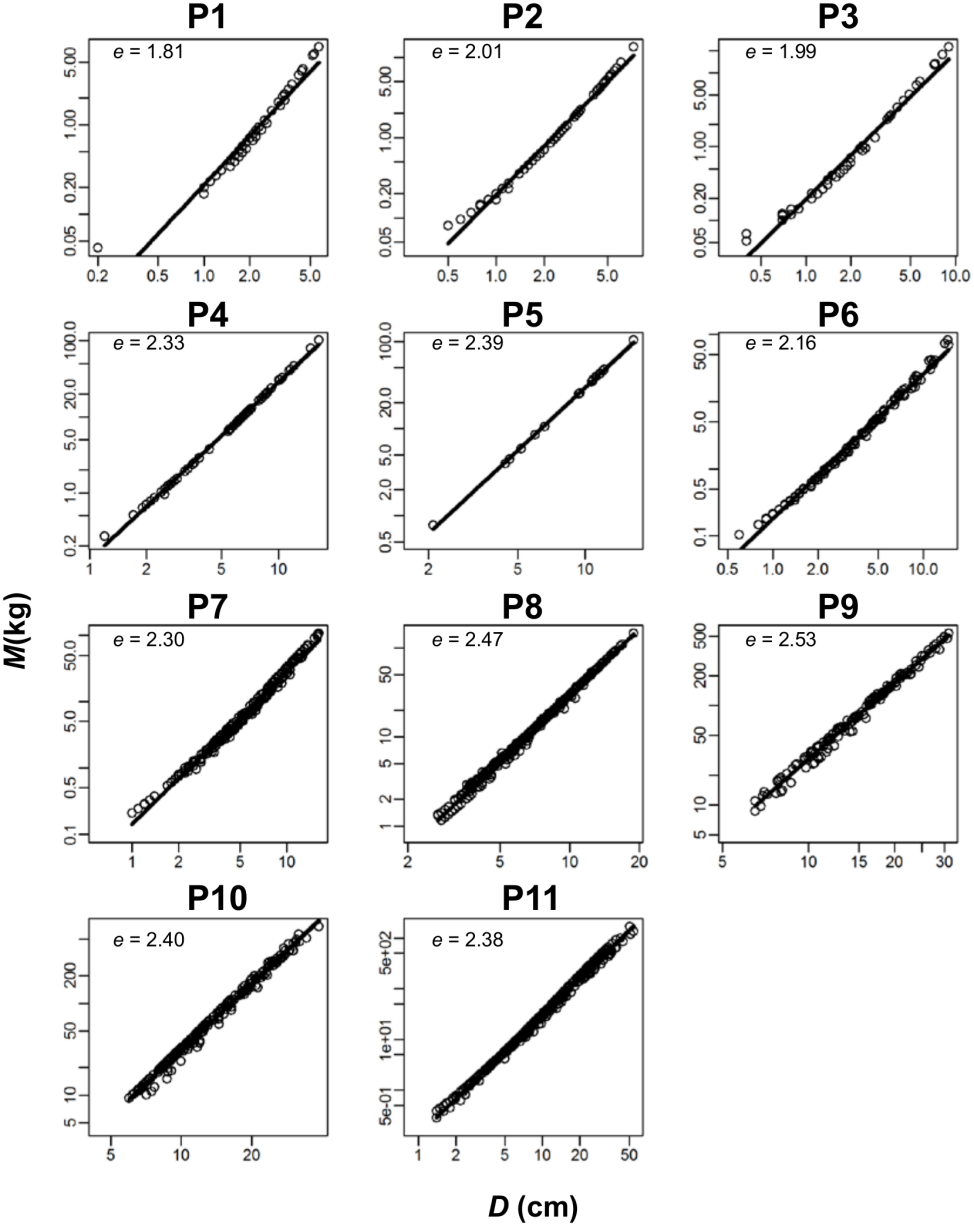
Allometric relationship between tree aboveground biomass (*M*) and tree stem diameter (*D*) in the last-year census. Shown are ln(*M*) = ln(*K*_4_) + *e* ×ln(*D*) fitted for the last year with estimated values of *e*. Plot 1 is the youngest and plot 11 is the most mature forest.

Estimated tree aboveground biomass in the 11 forest plots showed a scaling relationship to stem diameter (Fig 6). However, the 95% CI of scaling exponent *e* of *M* = *K*_4_*D*^*e*^ did not include 8/3, and the estimates were smaller than 8/3 (i.e., ≈2.67) in all years and plots (*e* = 1.71–2.54 by OLS regression, 1.74–2.55 by RMA regression). In particular, younger stands had a smaller scaling exponent (Fig 6; S1 Fig). Measured biomass of harvested dataset [27] also showed a scaling relationship to stem diameter. Again, the 95% CI of *e* was smaller than 8/3 (*e* = 2.33–2.36 by OLS regression, 2.35–2.38 by RMA regression). Therefore, assumption 6 was supported partially.

### Relationship between initial and final stem diameters

Eq (18) fitted well to the observed *D*_0_^2/3^ and *D*_T_^2/3^ data in both young and mature stands (Fig 1, black curve, parameter values are given in S2 Table). Eq (18) produced a curvilinear relationship between *D*_0_^2/3^ and *D*_T_^2/3^ from the obvious curve relationship in younger stands to the almost linear relationship in mature stands. The adjusted coefficient of determination was 0.64–0.99. In plots 1–7, Eq (18) explained 2–14% more variation in *D*_T_^2/3^ than Eq (3) by Enquist *et al*. [2] with a slope of unity. For plots 8–11, there was no difference in explained variation between two equations (0.0–0.8%).

### Relationship between stem diameter growth rate and initial diameter

**Fig 7:**
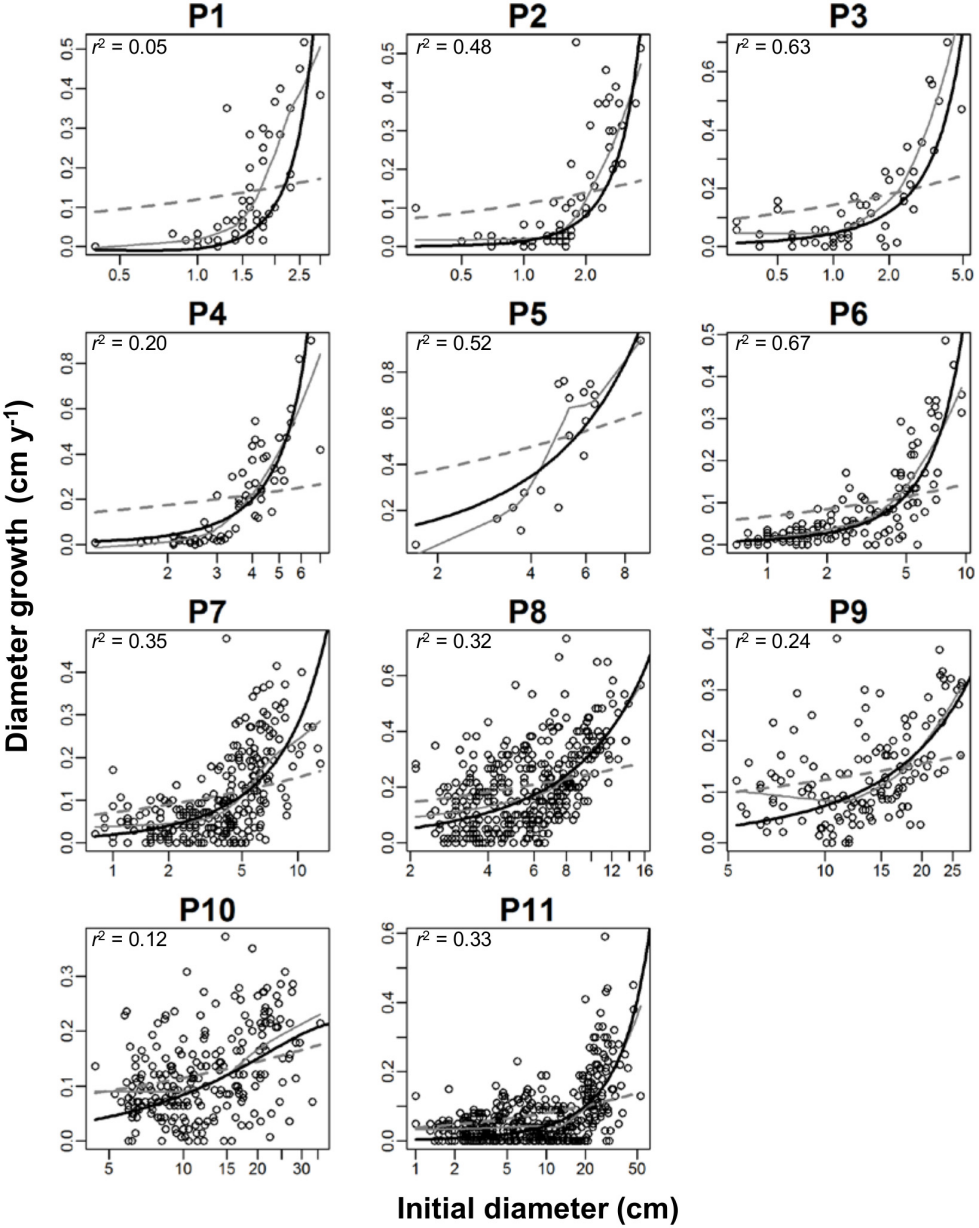
Relationship between initial stem diameter (*D*_0_) and diameter growth rate, (*D*_T_*—D*_0_)/*Δt*. Black curves are the proposed relationship expressed by Eq (19). Adjusted *r*^2^ values are shown. Parameter values are given in S2 Table. Dashed gray lines show the model of Enquist *et al*. [2], d*D*/*dt* = *βD*_0_^1/3^, fitted by non-linear regression. Gray curves are the LOWESS smoothed curve using locally weighted polynomial regression by the lowess function of the R software. Plot 1 is the youngest and plot 11 is the most mature forest.

Eq (19) fitted to the observed convex relationship between the initial stem diameter and diameter growth rate in all plots (Fig 7, black curve, adjusted *r*^2^ = 0.05–0.67, parameter values are given in S2 Table). The model of Enquist *et al*. [2] for tree growth rate (Eq 2) did not fit to the data (dashed line).

## Discussion

Our Eq (19) fitted to the observed convex relationship between growth rate of stem diameter and the initial stem diameter better than that of Enquist *et al*. [2] for all forests (Fig 7). Convex relationship between growth rate and size was shown to suggest asymmetric competition, while the linear-like relationship suggesting no competition or symmetric competition [7,29]. Indeed, asymmetric competition is the theoretical basis underpinning our model. Eq (18) was derived from Eq (4), which was mathematically derived from the growth model assuming asymmetric competition [17]. On the other hand, the model of Enquist *et al*. [2] is based on the assumption that tree growth is linear to leaf biomass. This means that both small and large trees have the same resource availability per unit leaf biomass. Such assumption may not be always true especially in closed forests where asymmetric competition for light is prominent than symmetric competition [6–8]. Smaller trees obtain less light resource per unit leaf biomass due to shading by larger trees resulting in reduced diameter growth than expected by the model of Enquist *et al*. (Fig 7). Thus, our results suggest that asymmetric competition is an important process in tree diameter growth.

The effect of asymmetric competition on tree growth can be seen in the difference between plots 4 and 5. Plot 5, which was adjacent to plot 4, was heavily thinned (95% of individual trees were thinned). The curve by Eq (19) was less convex in plot 5 than plot 4 (Fig 7). This suggests that smaller trees are less suppressed in their growth in plot 5 than those trees in plot 4, which may be the result of weakened asymmetric competition due to heavy thinning.

The model by Enquist *et al*. [2] and our model both fitted to the linear-like relationship beteween *D*_T_^2/3^ and *D*_0_^2/3^ in mature forests (Fig 1) even though two models are based on different ecological assumptions. This is because the linear-like relationship between *D*_T_^2/3^ and *D*_0_^2/3^ is a spurious trend resulting from autocorrelation. Since *D*_T_ = *D*_0_ + T∙d*D*/d*t*, the correlation between *D*_T_^2/3^ and *D*_0_^2/3^ should include an autocorrelation of *D*_0_ [30]. The degree of autocorrelation may be greater when the contribution of *D*_0_ to *D*_T_ is greater. In other words, the autocorrelation is stronger in mature forests, as shown in Fig 1. To avoid artifact due to autocorrelation, it may be better to analyze the relationship between diameter growth rate (d*D*/d*t*) and initial diameter (*D*_0_) rather than between *D*_0_ and *D*_T_. The theoretical relationship between Δ*D*/Δ*t* and *D*_0_ was easily obtained as Eq (19).

Our model is a non-spatial model that considers asymmetric competition. Various growth models that incorporated asymmetric competition have been proposed [6,8,12,31,32]. Some studies are based on sigmoid growth curve such as logistic model and Birch’s model [31,32]. However, there has been debate about the mathematical form of plant growth or even about sigmoid growth in trees [33–36]. Our model is not affected by the form of growth curve because we start from size frequency distribution (*Y*-*N* curve). Similarly, the growth model by Yokozawa & Hara [8] assumed no a priori growth function. Their model can evaluate the strength of symmetric and asymmetric competition from the field data. However, to apply to field data, spatial data of tree distribution was needed [37,38]. The models that extended the metabolic scaling theory [6,12] also required spatial data as well as data of light availability and crown area to parameterize the effect of asymmetric competition. Our model does not require these spatial, environmental, and morphological data and therefore may be applicable to various forest inventory data, which usually lack these detailed information.

Another characteristic of our model is that it deals with averaged growth rate for a given size. It cannot express the variation in individual tree growth due to local crowdedness as recent spatially explicit individual-based modeling can (eg. [39–41]). However, “macroscopic equation” like our model may be more useful for the future prediction of forest dynamics using dynamic global vegetation model [3].

Eq (19) described the curvelinear relationship of diameter growth rate to initial diameter (Fig 7). However, in some stands, the *r*^2^ value was not high. There may be some reasons to dilute the size dependency of diameter growth. We used the assumption that tree biomass scales to the 8/3 of diameter, but this relationship was not fully supported as shown by previous studies [42,43]. The growth rate may be different among species or growth form even if their size is same [12,30,44]. Because our model is based on asymmetric competition, our model may not perform well in forests where symmetric competition predominates or where interaction among individuals is weak. Examples of such forests may be the forests that have low tree density and crowdedness [38] or forests developed at low-productivity or high-disturbance sites [6,45]. Future researches are needed to improve our model or test the generality of our model.

## Supporting Information

S1 Fig Estimated scaling exponent *e of the allometric relationship between tree* aboveground biomass (*M*) and tree stem diameter (*D*) for each plot. Value of *e* in ln(*M*) = ln(*K*_4_) + *e* ×ln(*D*) fitted by OLS regression for each year and plot is shown. Error bars show the maxima and minima of each forest. Edges of the box show the upper and lower quartiles. Bar shows the median.
(TIF)

S1 Table Species composition of 11 plots. (CSV)

S2 Table Parameter values used to fit Eqs (18) and (19). (CSV)

S3 Table Tree growth data of 11 plots. Tree species name (Species), family name (Family), tree id (Tree.Number), diameter at breast height in year 19XY (DXY), tree height in year 19XY (HXY), and tree aboveground biomass in year 19XY (WAXY).
(ZIP)
