# Supplementary figures and images for: A New Model for Size-Dependent Tree Growth in Forests
Source: PLoS One. 2016 Apr 1;11(4):e0152219. doi: 10.1371/journal.pone.0152219 (PMC4817984; doi:10.1371/journal.pone.0152219)

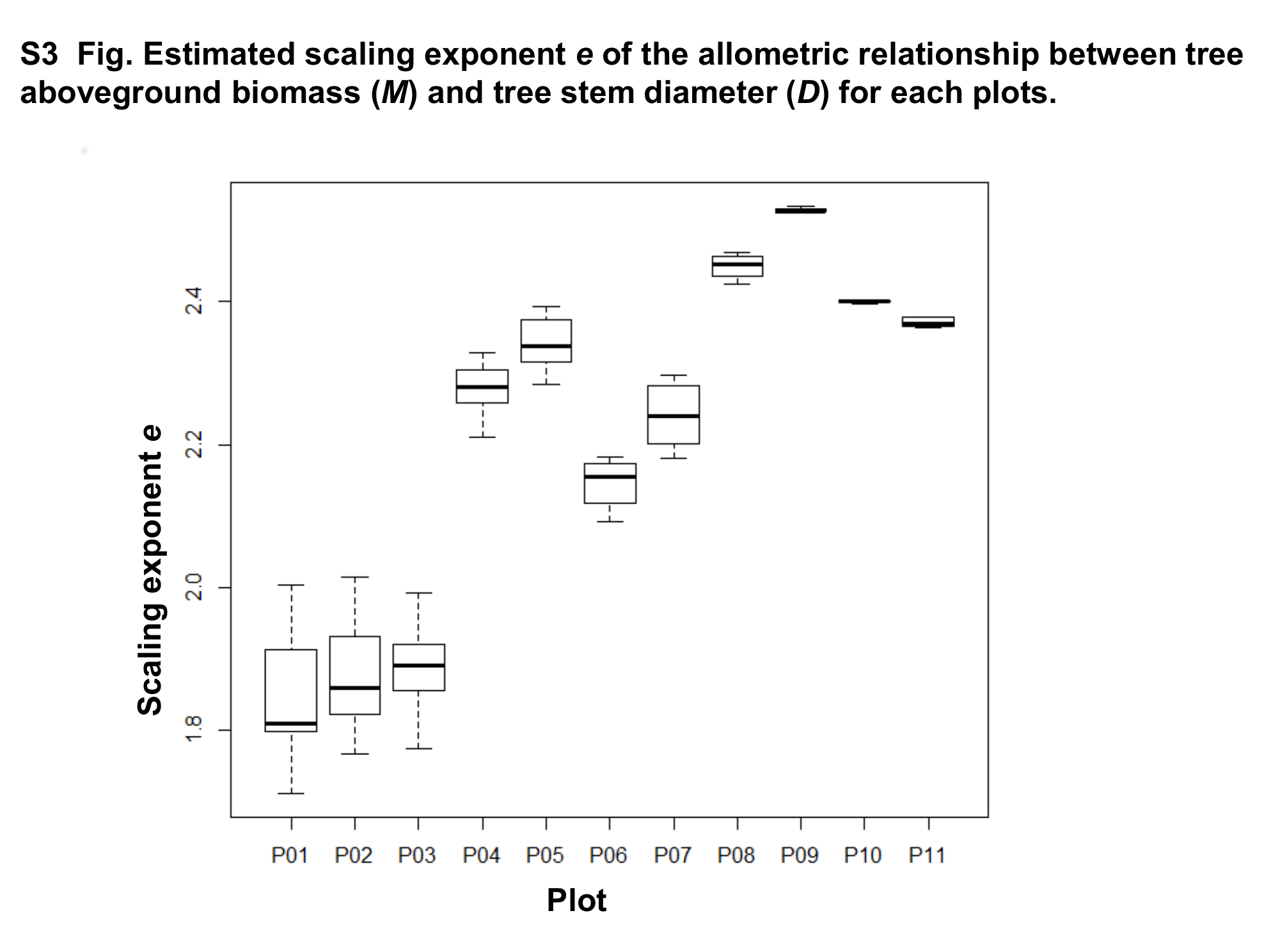

Supplement: S1 Fig — Value of e in ln(M) = ln(K4) + e ×ln(D) fitted by OLS regression for each year and plot is shown. Error bars show the maxima and minima of each forest. Edges of the box show the upper and lower quartiles. Bar shows the median. (TIF) [file pone.0152219.s001.tif]
